# Hospital and Institutional News

**Published:** 1918-09-14

**Authors:** 


### HOSPITAL AND INSTITUTIONAL NEWS.

### THE KING'S FUND FOR THE DISABLED.

THE KING has been pleased to give not only his name, but also the magnificent sum of £78,000 to the Fund which was recently founded to assist disabled officers and men of the Navy, Army, and Air Force. The trustees of the Fund, of whom Mr. John Hodge, the Minister of Pensions, is the chairman, appeal to every man and woman, indeed to children too, for a subscription. This will help to find not only a place in civil life for officers and men of His Majesty's Forces disabled in the war, but also benefit the widows and children of officers and men who have fallen. The Fund is established on the grounds that a State pension scale must be hard and fast, and that a State scheme must be a classification according to types. The King's Fund is to transcend classification, and to act, not as a public official, but as a private friend. The trustees report the receipt of 600 applications a week. So far 2,500 cases are understood to have been dealt with. Where the officer or man has been trained by the Ministry of Pensions, or where there is a business given up for war service, which he can restart, a grant can be made. The total aimed at is £3,000,000. The King has led with a Royal gift of £78,000. Who will follow the King's lead? We believe that there will be no hesitation or delay, but the trustees must also be warned that the generous response on which they rely is part of a larger impulse which will tolerate no stinting of grants, no bureaucratic officiousness, and no more of the delays and excuses from which other funds of the kind have not been free.

### THE DISABLED IN INDUSTRY.

PERHAPS the most interesting feature of the Conference of Local War Pensions Committees opened at Birmingham on September 10 (which the Pensions Minister refused officially to recognise because he had not been consulted before it was summoned) was the discussion on "The Disabled Man in Industrial Life," opened by the Hon. E. Cozens-Hardy. Another vital subject, wisely selected for debate, was the "Organisation and Functions of Local Committees." This debate, being more definite, and less general, should not evaporate into carbon-dioxide gas as do so many discussions. But the details are probably less important than the Conference itself. It is of course part of what may be termed an educational campaign to awaken the different cities to a lively sense of the problems ahead, and of their duty to the men who will be the chief part of that problem; to exchange experiences, to co-ordinate effort—in short, to tackle a national problem by national but decentralised means. It is probable that many men who have been patched up sufficiently to enter industrial life will relapse in one way or another into the care of the hospitals again, and since after the war the tendency will probably be to insist on a return to industry of these men too quickly, institutions are likely to find much more demanded of them than the arduous "training" which they supply at present.

### HOSPITALS ON THE ARMY MODEL.

COLONEL J. LYNN THOMAS, whose name has become familiar to the hospital world, has lately declared that hospital accommodation would be improved if it followed the lines of Army medical organisation. Since his recent expression of opinion in the newspapers that the ideal was for every class to have the right of admission to voluntary or public hospitals, he is reported to have given an interview to a correspondent of the *Newcastle Journal*, in the course of which he remarked on "the tremendous development which has been brought about in the British Medical Service" in France especially, where "those who are responsible are dealing with most complicated matters with thorough efficiency." He mentioned with pride a hospital which in addition to treatment provides 14,000 meals a day, and the tone of the speaker's remarks suggests an enthusiasm for size and vastness of organisation. We wonder how far this enthusiasm, though natural, is hasty, like many fresh impressions. We ask whether the immensity of the need has not necessarily blinded us all to anything but the fact that somehow it was all dealt with, and whether these immense organisations do not depend for their working upon the fact that they were occasioned by, and devised for, soldiers, and lastly, whether it would be either possible or desirable to regiment civilians in the same way. Colonel Lynn Thomas's views on these questions would be interesting to all who, unlike him, are not fresh from the absorption of the Front.

### THE LATE DR. SAUNDBY: AN HONOURABLE CAREER.

THE recent death of Dr. Robert Saundby, of Birmingham, in his seventieth year removes not only a distinguished consultant of long standing, but one of the few men who began life in a very different sphere from medicine. Born in 1849, he became a tea planter in his youth, and might have remained so had not the climate of the East proved fatal to his health. On returning home, a long voyage in those days, he made friends with a doctor on board who, in spite of Saundby's age, advised him strongly to begin the study of medicine. Since he had no other plans, he decided to do so, and entering at Edinburgh University, graduated M.B. in 1874. After becoming physician at the Royal Infirmary, Edinburgh, and later at the Royal Hospital for Diseases of the Chest, in 1876 he arrived in Birmingham, as pathologist to the General Hospital. In 1877 he graduated M.D. and began eight years' work as assistant physician. Having become in the meantime a member of the R.C.P. (his Fellowship dates from 1887), he was promoted in 1885 to the post of physician, which he held for twenty-seven years, and retired as consulting physician in 1912. Distinctions fell upon him; from 1896 to 1899 he was president of the B.M.A., and for many years he was professor of medicine to the Birmingham University. At one time he was president of the Birmingham Library. He was also a Lieut.-Colonel of the R.A.M.C. (Territorial Force), and both as a medical writer, who devoted also a book to professional ethics, and as a consultant, his reputation extended beyond the Midlands. Those, like ourselves, who knew him personally had regard and respect for his gifts and his judgment, and watched with interest the growth of his influence in Birmingham from the first. Of three sons, one has been killed in the war, and the two others are in the Army.

### DEATH OF A MEDICAL AUTHORESS.

To us who reviewed so recently Dr. Margaret Todd's volume on Dr. Jex-Blake, the death of Miss Todd is an unexpected event which gives cause for a genuine and not impersonal regret. This will be shared by the readers who knew of her works of fiction issued over the signature of Graham Travers. Under sixty years of age, Miss Todd early made good use of her experience as a medical student of the Edinburgh School of Medicine, and this was recognised in the popularity of "Mona Maclean, Medical Student," a book which helped to satisfy the curiosity of the hour in a subject of venomous controversy. It was probably the most humorous of her novels, and these were useful in "educating" public opinion in favour of the change for which Miss Todd and her heroine, Dr. Sophia Jex-Blake, worked strenuously. We hope that the interest aroused by the life of Dr. Jex-Blake may lead to a fresh study of the early work of fiction, which will come freshly to the many of the present generation who have not read a work of its date and character.

### A LEICESTER PHILANTHROPIST.

The death of Sir Herbert Marshall, of Leicester, removes from the commercial world of music a man of commanding personality. Regent House, in London, is the headquarters of the firm of Marshall and Rose, Ltd., of which Sir Herbert Marshall was the head. Through his influence the Leicester Philharmonic Society was formed in 1886. His career in Leicester was marked by a close association with the political, municipal, and social life of the borough. He received the honour of knighthood in King Edward's Birthday Honours in 1905. His municipal career covered a period of twenty-one years. He was Mayor in the Diamond Jubilee Celebration year 1906-7. The Victoria Diamond Jubilee Endowment Fund for the Leicester Royal Infirmary was the town's expression of thankfulness for a prosperous and peaceful reign, and under the influence of Sir Herbert Marshall a sum of £10,000 was raised and handed over to the Endowment Fund. He also helped to raise during his mayoralty £3,000 for the Indian Famine Fund. His entertainment of 40,000 school-children, and of the old people of sixty years of age and upwards, to celebrate the Diamond Jubilee reign is recalled with pleasure by the present generation of Leicester residents.

### THE ACHIEVEMENT OF CHARING CROSS HOSPITAL.

In the midst of the great new scheme, of which the Royal Free Hospital and the South London Hospital for Women are the centre, people are apt to forget the really heroic task which has been accomplished at Charing Cross Hospital under the leadership of Mr. George Verity, the chairman. Mr. Verity has issued from time to time certain pamphlets, all of which, except the latest, were frank confessions of the difficulties ahead. It is little more than a year since he issued one, the good news of which was limited to the statement that on the traditional mortgage of £85,000, £39,000 was still owing. Some eight months later this sum was raised when the hospital's deeds were once more returned to the Council. That this could be accomplished proved how much steady work had been continuously carried on, for Charing Cross Hospital has been the cripple of the London institutions. It has been famous for its deficiencies, and when people thought of its fine wards they thought of them wearily and said, "'Vaulting ambition doth o'erleap itself'; these buildings were a mistake. How much money is wastefully spent in charity!" The hospital had almost become a reason for not subscribing to charity. It was a monument of seeming failure, which could not even be pulled down without further expense. Mr. Verity and the war changed all this. In the autumn of 1914 the fine wards, which were suffering more from want of use than from the most regular occupation, were reopened to receive the wounded from all the Fronts, and the Government grant promised them a degree of solvency. By January of the present year the hospital at last confronted the world unencumbered. It was rich in its freedom from creditors, and poor only through want of endowment. This successful removal of a crushing burden was the fruit of the year's work, during which the balance of the building debt of £15,000 had been paid, a new mortuary, chapel, and theatre had been built or opened, new nurses' quarters had done something towards the proper accommodation of the staff, and the kitchens, heating and lighting services had been modernised. In addition, there had grown up a tuberculosis dispensary, a child-welfare clinic, and a clinic for venereal disease, while the vast mortgage debt had at last been liquidated. Mr. Verity attributes this achievement to the application of business methods to philanthropy. It is a modest attribution. The result gained has been to turn an example of mismanagement into an inspiration of hope, for no hospital was so crippled as Charing Cross, and now it is the hospital of all others in London to which the public may be most proud to subscribe. Much remains to be done, but the future now depends not so much upon an approved leadership as on the support of a well-rewarded body of subscribers.

### THE AMERICAN HOSPITAL ASSOCIATION AND THE WAR.

THE twentieth annual convention of the American Hospital Association will be held at Atlantic City during September 24-28. It is to deal mainly with the problems arising from the war, prominent among which is the situation produced by the absence of physicians on active service. The teaching staffs are being depleted, and the general efficiency of the Medical and Nursing Services is in peril. The standardisation of hospitals is expected to be discussed, and another important subject is the effect of the Federal Inheritance Law upon bequests to hospitals. The staff difficulty could be remedied, it is suggested, by abandoning what is called the "rotating medical service," by which two or more medical men serve for short periods, in favour of a single man to hold the appointment continuously. The present plan, in spite of the advantages which it offers in ordinary times, is held now to be against the national interest. The test of whether any medical man should be excused from war service is held to be his indispensability for the whole of the year. Pleas on behalf of part-time service, it is urged, should be ruled out. To ensure co-ordination and a settled policy on this and other points it is hoped that the convention will be exceptionally well attended.

### THE LARGER THANKS.

IN view of the shortage of staff the Southwark Board of Guardians wished to retain the services of Dr. A. R. Moore, now medical officer at their Newington institution, which has been converted into an infirmary for the aged and sick. But on their approaching the Central Medical Council with a view to his retention Dr. Moore informed the Guardians that he was anxious to take up a commission on September 23. As a fit medical man he felt that he ought to be engaged on war work. The Guardians have withdrawn their appeal. They have decided to send him a letter in appreciation of his patriotic attitude, coupled with their regret at losing his services. The Guardians' regret must be as sincere as the patriotism of Dr. Moore, and this lends peculiar value to their letter. To congratulate someone on doing an action which is inconvenient is an example of the larger kind of thanks.

### THE LIMIT.

A SERIOUS form of impersonation has been complained of lately—namely, the adoption of a wounded soldier's uniform by touts and impostors who represent themselves to be collectors on behalf of this or that hospital or charity. Unfortunately, the general public has not sufficient wit or conscience to see that the act of setting a wounded soldier to the task of collecting money would be in itself such an outrage as to damn any institution which adopted it. We regret to add that the practice has been tried here and there, but on each occasion abandoned in the face of strenuous and indignant opposition. To abuse a nurse's uniform is bad enough; to abuse the uniform of a wounded soldier is even worse. We wish we could add that it has been thus abused by impostors only, and never by hospitals at all.

### THE FUTURE OF MECHANICAL DIVERSIONS.

THE recreations now offered to the wounded man in hospital include a picture screen upon the ceiling, and the electrophone over his head. The cinematograph has come to him, and his ears, if not his eyes, are at the music-hall. In the same way he hears services on Sundays. The good side of these inventions is obvious enough, and the cost is not very great. The Electrophone Company is announced to have offered to instal its instrument in two wards of the Endell Street Military Hospital for £13, and to maintain them at an annual cost of £12. What a vista this opens up!

### THE COST OF "ASYLUM PATIENTS."

FOLLOWING the lead of Fr. Higley, the Stepney Board of Guardians has passed a resolution to call the attention of the Local Government Board to "the ever-increasing cost of the patients at the Sick Asylum," and to ask the Mile End Guardians to co-operate in bringing the matter to the notice of the authorities. The mover pointed out that since 1910 the call upon Stepney Guardians for the upkeep of the Sick Asylum had risen from £13,000 to £18,000, while the number of patients from Stepney had fallen from 222 to 145 in the same years. The cost had increased by one-third, while the number of patients from Stepney had been reduced by 30 per cent. This means in effect that the cost has doubled. Fr. Higley attributed the increase mainly to the rise in salaries at the Sick Asylum. It was time, he added, that the attention of the Local Government Board was called to the matter, and if the Mile End Guardians agreed to join in the protest it would probably be effective.

### THE REMOVAL OF GERMAN WOUNDED FROM NETLEY HOSPITAL.

A CROWD of several thousand workers in Messrs. Thornycroft's works at Woolston lately marched in protest to the Royal Victoria Hospital, Netley, under the influence of a rumour that British soldiers had been removed from their beds to huts to make room for wounded German officers. The procession, headed by a band of buglers, took advantage of the dinner-hour to demand an interview with the day surgeon. In fact two protest meetings were held, and eventually the officer in charge received the protest. The suggestions were denied, and the deputation was informed that every German in the institution was about to be removed on the following day, and the reports suggest that this assurance was wrung from the officer in charge to satisfy the deputation. The viler lay newspapers feed and foment the hysterical element on these occasions, and we can only wish for the authors of a well-intended demonstration that they will not be blessed by the press which draws its name and its circulation from the gutter.

### A NEW SANATORIUM FOR YORK?

THE Health Committee of York hopes that the City Council will authorise the use of a part of the Fairfield Estate for a sanatorium for adults, including discharged soldiers. The consent of the North Riding County Council has to be obtained, but the scheme is no new suggestion, and if adopted would absorb, apart from Raywell House and grounds of sixteen acres, thirty acres more. The rest of the land would, it seems, be sold to the North Riding Asylum Authority. Meantime the Health Committee is hard pressed to secure a suitable tuberculosis officer. Two applications for limited periods only have been withdrawn, and the only solution in view of the absence of candidates seems to be the recall from France of Dr. Bell Ferguson, who is now a commissioned officer.

### DISTINCTION FOR MR. GEORGE ROBEY.

IN acknowledgment of the assistance which he has given in opening a special appeal for the London Hospital, Mr. George Robey has been added to the list of life governors. He is one of the happiest examples of the co-operation between the theatrical profession and the hospitals, a co-operation which is to be found equally in the ranks of those who perform in the variety and, as Mr. Robey might call it, the monotonous theatre.

### A NEW THING IN ARCHITECTURE.

THE new Orthopædic Hospital for Service men at Tynecastle in connection with the Eastern District War Pensions Scheme is to be opened on October 5 by Sir A. Boscawen, the Parliamentary Secretary of the Ministry of Pensions. Intended for men who have been discharged from hospitals or for out-patients, the hospital has been designed by Mr. Robert F. Sherar, of 6 Ann Street, Edinburgh, and presents the interesting features of a unit or department, the need for which has been created by the war. In that sense it is a new thing in hospital architecture, the plans of which are worth study and attention.

### THE "MISCONDUCT RULES" OF APPROVED SOCIETIES.

APPROVED societies, which invariably have a rule prohibiting members to receive benefits when their bad health is due to venereal disease, are being asked to cancel this rule. This request is made by the National Health Insurance Commissioners, who draw attention to the special provision of the 1918 Act which facilitates the amendment of such a rule. The Royal Commission declared that the "misconduct rules" of approved societies seemed likely to deter patients from seeking the treatment that they need. As the Commissioners now say, these rules were for the most part adopted in the earlier days of the Friendly Society movement, when the serious indirect and later effects of these particular diseases and, still more, the recently discovered methods of their effective treatment were not known. There is a widespread feeling that the refusal of benefit to those suffering from any form of venereal disease is not only prejudicial to the health of the community and contrary to the national interest, but may also, by discouraging early and proper treatment, in the long run prove injurious to the funds of societies.

### DUTCH SOCIAL WORK IN CONNECTION WITH NERVOUS DISEASE.

A DUTCH correspondent writes: Under the high direction of the Royal Medical Inspector of the Dutch Asylums, Dr. J. H. Schuurmans Stekhoven, Knight of the Dutch Lion and Fellow of the Dutch Central Health Council, there has been started this year an "association for social work in connection with nervous disease and insanity." This new society has promptly and successfully called for the moral and material aid of the Dutch Government and various other civilian authorities, and with their help established at 27 Schoolstraat, Utrecht, a central office, bearing the name of "Orion," mindful of the proverb "A good beginning is half the battle." It may be said that this new association supplies a felt want, for social work in Holland, especially in connection with nervous disease and insanity, has up to this time been neglected.

### THIS WEEK'S DRUG MARKET.

A DISTINCT improvement in business is noticeable, and on the whole prices are well maintained, while several articles have a strong tendency to move upwards in value. Italian produce—as, for instance, lemon oil, bergamot oil, and citric acid—is dearer; lemon oil especially is inclined to advance in price. Menthol continues to attract attention, and a good business has been done at the advanced rates. Seidlitz powder and tartarated soda are dearer. The demand for camphor tablets continues large, and English refiners state that their output is booked up for several weeks ahead. Acetyl-salicylic acid is dearer, but phenacetin still has a downward tendency in price. The position of bromides is unchanged except that makers are offering small quantities of ammonium bromide to their regular customers at lower rates. Cascara sagraada is dearer. Makers of glycerophosphates state that owing to the impossibility of obtaining further supplies of glycerin they will be unable to accept orders after their present stocks are exhausted. The price of honey is firmly maintained. The position of saccharin is unchanged. Sugar of milk is offered at lower prices, and it is expected that maximum selling prices will shortly be fixed.

### TO OUR READERS.

**CONTRIBUTIONS are specially invited from any of our readers to these columns. They should deal with topical subjects and news. They must be authenticated for the information of the Editor only. The minimum payment if published is 5s. There is no hard-and-fast rule as to space, but notes of about twenty lines in length are preferred.**

